# Chest circumference in full-term newborns: how can it be predicted?

**DOI:** 10.1186/s12887-019-1712-3

**Published:** 2019-09-26

**Authors:** Ingrid G. Azevedo, Norrara S. O. Holanda, Nivia M. R. Arrais, Raweny T. G. Santos, Ana G. F. Araujo, Silvana A. Pereira

**Affiliations:** 1 Ana Bezerra University Hospital - Empresa Brasileira de Serviços Hospitalares, Federal University of Rio Grande do Norte (UFRN), Praca Tequinha Farias, 13, Santa Cruz, RN 59200-000 Brazil; 2 Faculty of Health Sciences, Federal University of Rio Grande do Norte (FACISA – UFRN), Rua Teodorico Bezerra, Santa Cruz, RN 59200-000 Brazil; 3 Department of Pediatrics, Federal University of Rio Grande do Norte (UFRN), Campus Universitário Lagoa Nova, Natal, RN 59078-970 Brazil; 4 Graduate Pediatrics and Applied Sciences in Pediatrics Program, Federal University of São Paulo (UNIFESP), São Paulo, SP Brasil; 5 Postgraduated Program in Rehabilitation Science - FACISA - UFRN, Santa Cruz, Brazil; 6 Department of Physical therapy and Postgraduated Program in Rehabilitation Science - FACISA, Federal University of Rio Grande do Norte UFRN, Campus Universitário Lagoa Nova - CEP 59078-970, Natal, RN Caixa Postal 1524 Brazil

**Keywords:** Chest circumference, Newborns, Development, Predictive equation

## Abstract

**Background:**

Although over the years a number of studies have used chest circumference (CC) as a sensitive tool to identify the health status of infants, a particularly important aspect for this population is the lack of data on normal values and prediction equations. In order to facilitate and validate the interpretation of CC data in newborn (NB), the aim was to study the relation between CC and other anthropometric variables and develop a predictive equation for CC in a population of full-term newborns.

**Methods:**

Cross-sectional study, carried out with full-term infants. The anthropometric (CC, head circumference - HC, length, age and weight) and hemodynamic variables were evaluated during the first 24 h of life. Bivariate analysis was performed between CC and HC, weight, length and type of delivery, followed by multiple linear regression analysis, including variables that were significant in the bivariate analysis. For data analysis, we used the SPSS program, considering *p* < 0.05 and 95% CI.

**Results:**

The birth weight of the 120 NB varied between 2580 and 4225 g (mean 3360 g) and the gestational age between 37 and 42 weeks (mean 39 weeks). Approximately 61% of the sample were delivered vaginally and 67 (56%) were boys. The variables that remained statistically associated with CC after multivariate analysis were weight (β 0.003, CI: 0.002: 0.003, *p* = 0.001) and HC (β 0.287, CI: 0.156: 0.417, *p* = 0.001). For the linear regression model, the predictive equation of CC was 14.87+ (0.003 x weight) + (0.287 x HC), with a prediction of 76%.

**Conclusion:**

The results show a positive correlation between CC and weight, length and HC, and based on the linear regression model, the predictive equation for CC is based only on weight and HC.

## Background

Assessment in perinatology uses anthropometry as an essential tool to monitor growth and evaluate the nutritional and functional status of newborns (NB). It is an economical, non-invasive and easy-to-execute tool that can improve understanding of growth patterns and their variations [1].

Studies in different populations suggest the use of anthropometric parameters is an important substitute for early identification of low-weight newborns [2–4]. Of these variables, head circumference (HC), chest circumference (CC) and abdominal circumference (AC) exhibit good correlation with the health status and well-being of children and are widely used as indicators of health, performance and survival [5, 6].

CC, HC and AC are simple, reliable and logistically viable tools in field conditions that can be used on a large scale in both domiciliary care and high-complexity environments due to their accessibility. These resources, whose aim is early detection of developmental changes and prevention of newborn deaths [2], are important methods, capable of predicting the need for early referral of low-birth-weight babies for extra care, given that after surviving the critical neonatal period, they may experience compromised physical and mental growth [2].

In a study involving NB hospitalized in a neonatal intensive care unit (NICU), Hadush, Berhe & Medhanyie, 2017 [7] demonstrated that birth weight showed a strong correlation with anthropometric variables (CC and HC). In this study, conducted in Ethiopia, CC and HC were used to identify low-birth-weight newborns. The cutoff points for better sensitivity and specificity identified in the study were 30 cm and 31 cm for CC and HC, respectively, the former being the best predictor of low birth weight [7].

Asian studies also showed CC < 30 cm and arm circumference < 8.7 cm, with better sensitivity and specificity in identifying low-birth-weight NB [2, 8]. Alternative measures to identify low birth weight have been recommended by the World Health Organization (WHO) [9] since the last decade. Furthermore, it is important to consider that CC is easier to measure, because the nipple line can be clearly and objectively established as a reference point, making its identification operationally feasible when compared to HC, or even a scale to estimate weight [7].

Although over the years a number of studies have used CC as a sensitive tool to identify the health status of infants, establishing a good reference for clinical practice in neonatology [10, 11], a particularly important aspect for this population is the lack of data on normal values and prediction equations.

Thus, in order to facilitate and validate the interpretation of CC data in NB, the aim was to study the relation between CC and other anthropometric variables and develop a predictive equation for CC in a population of full-term newborns.

## Methods

This is a cross-sectional observational study, conducted at Ana Bezerra University Hospital, located in Northeastern Brazil, between April and November 2017. The medical-assistance service, which provides dedicated prepartum and postpartum care for the mother-infant dyad promotes maternal-infant bonding, where both remain close to each other and are cared for by a multiprofessional team. There are 51 licensed beds, 9 delivery rooms and the postpartum nursery can accommodate up to 32 beds. The Neonatal Intensive Care Unit (NICU) provides quality care for high-risk newborn infants free of charge and can accommodate up to 10 beds. The neonatal team consists of 10 staff physicians and 4 rotating pediatric residents, 10 nurses, 4 physical therapists, 3 nutritionists, 2 speech therapists and 7 multiprofessional residents.

A convenience sample of 151 NB was recruited, based on the adequate gestational age (GA) (37 to 42 weeks) [12], of both sexes, at up to 23 h of life, spontaneous breathing full-term NB, hemodynamically stable and exhibiting no chest deformities. We have used WHO child growth pattern, which shows physical growth curves, achievement windows and performance milestones [12]. In this sense, the z-score of height for age was considered, considering the positive (+) or negative (−) value depending on whether the child’s real height is higher or lower than the median height of the WHO reference population at that age for that sex. Children with values found within the median of the WHO reference population were considered for the study.

The anthropometric variables birth weight, CC, HC and length were measured according to the Brazilian Pediatric Society’s Orientation Manual [13] and corroborating the methodology described in studies by HADUSH; BERHE; MEDHANYIE, 2017 [7]. The measures of CC and HC were obtained with a non-stretchable tape measure while NB were in the supine position; to assess CC, the tape was wrapped around the head midway on the occipital bone, and for HC, the tape was wrapped under the axillary area around the back to meet at the mid-sternal area at the nipple level.

The hemodynamic variables heart rate (HR), systolic (SBP) and diastolic blood pressure (DBP) were measured using a DIXTAL DX 2022® multiparametric monitor with the NB lying on a bed with a suitable cuff (width corresponding to 2/3 of arm length, extension to ½ of its diameter) placed on the left arm. Respiratory rate (RR) was measured by observing chest expansion, counting the number of breaths per minute. The 1 and 5-min Apgar scores and type of delivery were recorded on the medical chart. All the variables were assessed during the first 24 h of life in the company of the newborn’s mother or guardian.

The data collected were analyzed using the Statistical Package for the Social Sciences (SPSS) program, version 21.0 (SPSS for Windows, Chicago, USA). The Kolmogorov-Sminorv test was applied to determine data normality. Descriptive statistics for all the variables were expressed as mean and standard deviation, according to sex, and analyzed using the Mann-Whitney test for quantitative variables and the chi-squared test for categorical variables. Bivariate analyses were carried out applying simple linear regression. The multiple linear regression model was used to determine the correlation between CC and the significant covariables in the bivariate analysis (*p* < 0.05). For all the stages, we considered p < 0.05 and CI 95%. Moreover, regression diagnoses were conducted for all linear analyses, including collinearity tests, with no significant deviations.

## Results

A total of 151 NB were assessed and 31 were excluded due to sleepiness and persistent crying. The final sample consisted of 120 NB, with birth weight varying between 2580 g and 4225 g (mean 3360 g) and GA between 37 and 42 weeks (average of 39 weeks). Approximately 61% of the sample were born vaginally and 67 (56%) were boys. There was no statistical difference between hemodynamic measures and RR, or type of delivery, when the sample was considered separately by sex, as shown in Table 1. However, weight, length, HC and CC were higher in the boys, exhibiting a statistically significant difference.

**Table 1:** Newborns characteristics (*n* = 120), Santa Cruz - Brazil - 2017

	Boys (*n* = 67) n (%)/Mean (SD)	Girls (*n* = 53) n (%)/Mean (SD)	*p*-value
Weight (g)	3360.22 ± 365.36	3142.24 ± 356.82	0.003^a^
Length (cm)	49.18 ± 2.20	47.67 ± 2.21	< 0.001^a^
GA (weeks)	39.20 ± 1.20	39.05 ± 1.35	0.428^a^
HR (bpm)	126.40 ± 16.19	129.88 ± 20.70	0.564^a^
RR (brpm)	48.71 ± 9.01	46.33 ± 8.89	0.223^a^
SBP (mmHg)	122.58 ± 17.29	126.45 ± 21.34	0.248^a^
DBP (mmHg)	47.01 ± 10.42	45.83 ± 9.07	0.682^a^
CC (cm)	34.13 ± 1.46	33.51 ± 1.39	0.036^a^
HC (cm)	34.80 ± 1.35	34.18 ± 0,89	0.006^a^
Type of delivery			0.157^b^
Vaginal	37 (55.2)	36 (67.9)	
Cesarean	30 (44.8)	17 (32.1)	

*a* Mann-Whitney, *b* Chi-squared, *g* gram, *cm* centimeter, *bpm* beats per minute, *brpm* breaths per minute, *mmHG* millimeters of mercury, *GA* gestational age, *HR* heart rate, *RR* respiratory rate, *SBP* systolic blood pressure, *DBP* diastolic blood pressure, *CC* chest circumference, *HC* head circumference

Table 2 shows the bivariate analysis between CC and the following variables: type of delivery, weight, length and HC. There was no statistical difference between CC and type of delivery, but it was significant for length, weight and HC.

**Table 2:** Bivariate analysis of newborns chest circumference values according to sample characteristics (*n* = 120), Santa Cruz - Brazil - 2017

	β (CI95%)	*p*-value
Type of Delivery	−0.094 (− 0.591: 0.403)	0.708^a^
Weight	0.003 (0.002: 0.003)	< 0.01^a^
Length	0.300 (0.199: 0.400)	< 0.01^a^
Head Circumference	0.764 (0.634: 0.895)	< 0.01^a^

^a^simple linear regression

When the significant bivariate analysis variables were included in the multiple linear regression analysis, only weight and HC remained as predictors of CC, as exhibited in Table 3.

**Table 3:** Multiple linear regression model for variables associated with newborns CC (*n* = 120), Santa Cruz - Brazil - 2017

Variables	Adjusted β (CI 95%)	*p*-value
Weight (g)	0.003 (0.002: 0.003)	< 0.001
Head Circumference (cm)	0.287 (0.156: 0.417)	< 0.001

*g* gram, *cm* centimeter, *CI* confidence interval

From the regression model, the predictive equation of CC is 14.87 + (0.003 x weight) + (0.287 x HC).

## Discussion

Monitoring and assessing NB development is essential to promoting health and preventing complications, thereby guaranteeing higher newborn survival rates [14, 15]. Healthy growth is reflected in a physical increase in body size, measured in centimeters or grams, and results in a rise in the number and size of cells and in their degree of cellular differentiation, for the development and execution of organic and functional activity [16]. These events occur simultaneously to preserve and maintain harmonious relations at the same magnitude and proportion of brain, spine, rib cage, and limb development, in addition to other body segments [17, 18].

The circumferences measured separately in the present study are within normal ranges [12], showing a positive correlation between CC, length and HC, regardless of sex, with weight and HC as the main predictors of CC.

In the present study, the newborns exhibited adequate weight for gestational age, and satisfactory HC and CC, with higher measures in the group of boys. However, the difference in CC between the girls and boys was not statistically significant, and similar to normal growth patterns reported in the literature [6].

In a longitudinal study, Jaldin et al. [10] assessed CC growth in exclusively breastfed children in their first 6 months of life. These authors obtained higher CC values in boys, albeit not exceeding 1 cm above the average for the age under study, corroborating the present study. Breastfeeding is very important for newborns to ensure adequate CC growth, as well as routine monitoring of children aged between 0 and 24 months, the period of greatest post-natal growth [10, 11, 13–23].

Streja et al. [24] conducted a study on the weight/CC ratio at birth and assessed NB growth according to maternal factors in the intrauterine period of fetal development, observing body disproportionality in newborns that were small for gestational age, in terms of weight, CC and AC. These measures are considered developmental markers, based on the condition of the newborn, considering that the mother’s condition and her life habits are determinants of neonatal growth [25, 26].

From this standpoint, it is important to underscore each child’s individual development pattern, which consists of an intrinsic relationship between the newborn’s intrinsic and extrinsic factors, since the infant undergoes a series of transitions from intrauterine to extrauterine life. In this perspective, a contextualized assessment of the level and quality of NB development and formulation of predictive equations are indispensable [27–30].

The practical utility of the equation for neonatologists may be useful. Thoracic cavity abnormalities are usually detected in the neonatal period and often lethal [31] when they result in pulmonary hypoplasia. In these cases, HC or weight is not enough. This is because they are often associated with a small, narrow, elongated rib cage typical of abnormal rib development [32]. In this sense, the usefulness of detecting pathological CC as a spy of the chest malformation and/or as a dysmorphic feature may be important, if associated with clinical signs or in addition to other dysmorphic elements.

Another study [7] that discussed predictive equations involving CC investigated low-weight NB that were still hospitalized. The authors aimed at developing a simple, inexpensive and practical method to identify low-weight NB. Thus, the cutoff point used to detect low-weight NB would be 30.15 cm for CC and 33.25 cm for HC. However, given the better sensitivity observed, the measure of CC would be superior in detecting low-weight NB [7].

Bishnupada et al. [8] used anthropometric parameters, such as mid-upper-arm circumference, CC and HC, to identify their relation with birth weight in 316 NB in Dhaka, Bangladesh. The authors obtained an average birth weight of 2.889 g, corresponding to 15.18% of low-weight babies (< 2.500 g). Thus, sensitivity and specificity were considered better for CC, which is the best detector of birth weight, with a correlation coefficient slightly above 0.84 when compared to the other anthropometric measures. In an observational study, Ramagopal Shastry, Poornima & Bhat [1] also investigated the relation between birth weight and HC, and arm circumference and CC, with findings similar to ours.

In three regions of Himachal Pradesh, in India, a study was conducted to obtain a standard reference criterion (mean ± SD) for normal full-term NB parameters and the correlation between birth weight and HC, CC and AC. Chest circumference was 31.1 cm in boys and 31.0 cm in girls, corroborating the findings of our study. Furthermore, CC showed a high correlation (r = 0.752) with AC, compared with other anthropometric parameters according to birth weight. It was also observed that CC and AC showed greater correlation (r = 0.785) in Himachal Pradesh, (r = 0.752) the outer Himalaya and (r = 0.848) and the middle Himalaya. However, these findings exhibit some limitations, related to differences in genetic, nutritional and environmental factors when compared to the Brazilian population [33].

It is important to underscore that the high rate of cesarean deliveries in the present study is associated with insufficient dilation for natural childbirth, poor baby positioning and insufficient uterine dynamics. Cesarean sections are prevalent in all parts of Brazil, accounting for approximately 50% of the babies delivered in the country [34]. The percentage in the study sample is a reflection of what is occurring throughout the country. According to the World Health Organization (WHO) cesarean deliveries should not exceed 15%, and natural childbirth should be strongly promoted, with a focus on humanized care [34, 35].

In this respect, it is important to underscore the importance of the association between anthropometric variables in assessing child development in order to construct reliable indicators, corroborating the findings of the present study. Ndu et al. [36] took anthropometric measures (CC and HC) to detect low birth weight among NB from the Igbo people of Nigeria. These authors also found CC to be the best predictor to detect low-birth-weight babies, indicating that CC is a strong marker of development.

It is important to emphasize that variations in CC have been little studied in Brazil. Pediatric health care teams need more information on changes in CC, establishing the predictive equation, and standardizing measures to assess a child’s developmental progress, in order to identify the responses to extrinsic factors related to nutrition, growth and development.

In this respect, studies that provide a more detailed description of these measures, comparability and, especially, research involving interventions aimed specifically at improving nutritional and motor indicators should be encouraged. It is suggested that the CC percentiles of healthy term newborns be calculated in the future.

The present study exhibits limitations. For example, the sample size is considered small for the type of study, showing the need for new investigations with larger sample sizes, as well as including premature newborns.

## Conclusions

There is a positive correlation between CC and weight, length and HC. However, after adjusting for covariables, only HC and weight predicted CC. It is important to underscore the importance of knowledge regarding the main aspects related to the development and growth of NB. As such, neonatal and pediatric health professionals should consider these measures in order to provide comprehensive care to children, who should also be periodically monitored.

## Data Availability

The dataset used and analyzed during the current study is available from the corresponding author on reasonable request.

## Abbreviations

AC: Abdominal circumference
CC: Chest circumference
CI: Confidence interval
DBP: Diastolic blood pressure
GA: Gestational age
HC: Head circumference
HR: Heart rate
NB: Newborn
NICU: Neonatal intensive care unit
RR: Respiratory rate
SBP: Systolic blood pressure
SPSS: Statistical package for the social sciences
WHO: World Health Organization
